# Training in clinical research within individual health organizations: a strategic tool devised for enhancing predictive, preventive and personalized medicine within a comprehensive participative framework

**DOI:** 10.1186/1878-5085-5-S1-A157

**Published:** 2014-02-11

**Authors:** Guglielmo M Trovato

**Affiliations:** 1 Dipartimento di Scienze Mediche e Pediatriche – Unità di Terapia e Diagnostica non Invasiva, AOU Policlinico-VE, University of Catania, Italy

## 

We report our experience in a University Hospital (2012-2013) and in the School of Medicine (2001-2013) in courses committed to promote original thought, to provide basic and operationally useful skills, to teach and to explore the possibilities of setting up networks and existing collaborations. The contribution of ideas, resources, skills and tenacity is the basis for strategic researches aimed at promoting quality, effectiveness and efficiency of health systems. The specific environments and healthcare organizations for which the research is a structural mission need quality and visibility, indicators of quality and efficiency. Vocational training integrated and participated within all the active components with a clinical profile (doctors, nurses, technicians, biologists, dietitians, physiotherapists, others) will facilitate those who wish to plan projects also with international European profile [1,2]. The interaction and contribution of participants are the core of the training methodology and research is developed as a permanent mode of professional development. The course is intended as a modality of updating professionalism and expertise in the field of integrated health care organizations. It has a modular structure and enhances the articulation of medical and surgical areas with the services committed with the evaluation of the quality and with preventive and occupational medicine. The course is based on the enrichment of independent thinking for challenging established concepts and ideas, using also the reappraisal of already accomplished projects [3-5].

1) Actual research ongoing in the individual clinical institutions, by the presentation of projects implemented or under way, regardless of specific funding.

2) Strategies of Project Management through an analysis of the context (scenario), feasibility, impact and contingency plans.

3) Realistic prospects for fundraising, public and private, building-up overviews of applied research with the contribution of all students. The course implements the explicit use of methodologies of data-mining and the subsequent elemental analysis of usable information:

a) predictive models,

b) assessments of sensitivity/specificity (diagnostic tests),

c) comparative analysis of the risks in case-control or randomized studies, and the calculation of the required number,

d) comparison of averages or changes of measures in sequential studies,

d) definition of some possibilities for translational research with the development of models.

## Recommendations

The expertise in clinical research is limited in most institution and not fully oriented toward Predictive, Preventive, and Personalized Medicine (PPPM) concepts and goals. The consequence is that most funded research are addressed to innovative fields but show often limited attention to a shared patient-physician center strategy, to integration and even to outcome. These trainings to be comprehensive and effective must move along PPPM directives; these must be included in the projects that should be independently supported (such as EU funding) within competitive calls. The goal is overcoming the gap among predictive, preventive and personalized medicine training professionals – not only MDs -, for sustainable and rewarding research projects, including nutrition and lifestyle chance strategies by health psychology tools. The promotion of the professional quality in translational research (from epidemiology to personalized therapy) is the pre-requisite for innovative best practices and their dissemination of the most high impact information.
